# Prognostic Value of lncRNA H19 in Gastric Cancer: A Systematic Review and Meta-Analysis

**DOI:** 10.3390/ijms27209276

**Published:** 2026-10-19

**Authors:** Silu Song, Yi Li, Chenxia Gao, Shuai Li, Shaojing Jin, Shuang Liu

**Affiliations:** Key Laboratory of Microecology-Immune Regulatory Network and Related Diseases, College of Basic Medical, Jiamusi University, Jiamusi 154007, China

**Keywords:** lncRNA H19, gastric cancer, prognosis, meta-analysis

## Abstract

LncRNA H19 is a crucial regulatory player in GC and could operate as an attractive biomarker for patient prognosis. This meta-analysis aims to gauge the link between lncRNA H19 expression levels and patient prognosis, as well as clinicopathological features. HR and 95%CI were utilized to evaluate the connection between lncRNA H19 expression and prognosis, as well as clinicopathological features in GC patients. Bioinformatics analysis, potential molecular mechanisms and drug therapy involving lncRNA H19 were predicted through databases. The expression of lncRNA H19 in GC cells was verified through qRT-PCR. High expression of lncRNA H19 is correlated with a lower overall survival rate in GC patients, as well as TNM stage, lymph node metastasis and tumor invasion. Bioinformatics analysis confirmed that lncRNA H19 is significantly expressed in GC and related to poor prognosis. Function prediction analyses suggested that lncRNA H19 may contribute to GC progression through ceRNA regulatory networks, TFs, and predicted interactions with RBPs. Nutlin-3, PD-0332991, and Topotecan were predicted as preliminary candidate drugs. LncRNA H19 was confirmed to be highly expressed in GC cells. LncRNA H19 might act as a feasible biomarker for prognostic prediction in GC, warranting further validation in clinical studies.

## 1. Introduction

Gastric cancer (GC) is one of the most common malignancies of the digestive system, ranking fifth in global cancer incidence. It is characterized by high malignancy and late diagnosis, rendering it a predominant cause of cancer-related fatalities globally [1,2]. Annually, more than one million new cases are identified, and GC exhibits high heterogeneity. Early clinical symptoms vary widely among patients, contributing to poor prognosis and reduced quality of life [3,4].

The worldwide burden of GC is projected to rise by 62% until 2040 [5], mainly due to the aggressive invasion and metastasis observed in advanced-stage patients [6]. Substantial advancements have been achieved in the diagnosis and treatment of gastric cancer, aided by advanced technologies and molecular approaches. These include deep convolutional neural network real-time diagnostic system [7], endoscopic submucosal dissection [8], endoscopic ultrasonography [9], automatic nnU-Net combined imaging omics [10], and respiratory analysis and genomic analysis, such as circular RNA (cricRNA), long non-coding RNA (lncRNA), microRNA (miRNA), and other circulating biomarkers. In recent years, lncRNAs have become known as pivotal regulators in multiple diseases, bringing insights into intricate molecular mechanisms. There has been a growing emphasis on their significance in GC diagnosis, treatment, and prognosis, with the goal of enabling more precise and personalized therapeutic strategies.

Currently, more than 100,000 lncRNAs have been cataloged in public databases [11]. Among them, lncRNA H19 (H19) is one of the earliest reported, with a total length of 2.3 kb, situated on human chromosome 11p15.5. H19 is notably expressed throughout embryonic development [12] and functions as a multi-functional lncRNA, with its dysregulation closely associated with various human malignancies [13]. Increased evidence have highlighted the value of H19 in GC. Zhang et al. [14] noticed that H19 is increased in GC tissues and cells, promoting migration and metastasis via the ceRNA axis miR-148-3p/SOX-12. Liu et al. [15] discovered that high H19 expression is associated with worse prognosis in GC patients, causing to the nuclear translocation of β-catenin, activation of the Wnt/β-catenin signaling pathway, and facilitation of epithelial–mesenchymal transition (EMT), thus encouraging metastasis. Wei et al. [16] clarified that H19 influences GC progression via the let-7c/HER2 ceRNA axis. Chen et al. [17] identified that let-7a acts as a molecular sponge for H19, which in true targets COPB2 mRNA, promoting GC development via the H19/let-7a/COPB2 axis; treatment with the small-molecule compound Chrysin exhibited anti-tumor effects. Yu et al. [18] observed elevated H19 levels within the serum of GC patients and proposed H19 as a diagnostic and prognostic biomarker by combining clinicopathological data. Their study indicated that H19 promotes GC invasion via the miR-138/E2F2 pathway. Li et al. [19] found that H19 plays a role in tumor immunity. It regulates immune cell infiltration in GC via the miR-378a-5p/SERPINH1 ceRNA axis, identifying a prognostic immune-related pathway. Regarding immune escape, Sun et al. [20] found that H19 knockdown in GC cells reduced lactic acid synthesis via the miR-519d-3p/LDHA ceRNA axis, thereby inhibiting immune cell activity. H19 knockdown also suppressed aerobic glycolysis, disrupted the energy supply, and suppressed tumor cell proliferation, providing a basis for further studies on cancer metabolism. Similarly, Chen et al. [21] demonstrated that the H19/miR-19a-3p/phosphoglycerate kinase 1 axis regulates aerobic glycolysis in GC cells. Knockdown of H19 dropped extracellular acidification rates without significantly affecting oxygen consumption rate, indicating inhibition of aerobic glycolysis and reduced proliferation.

To date, H19 has shown potential as a prognostic biomarker in GC, with accumulating evidence linking its upregulation to poorer patient outcomes. However, the available studies remain fragmented and vary in sample size, sample type, detection method, and cut-off value. We therefore performed a meta-analysis to quantify the associations of H19 expression with survival and clinicopathological characteristics in GC patients. Independent UALCAN and GEO datasets were used as complementary analyses to examine whether H19 overexpression and its associations with survival and TNM stage could be reproduced in external data, while qRT-PCR assessed H19 expression in GC cell lines. In contrast, GO and KEGG enrichment, ceRNA network construction, transcription factor and RNA binding protein prediction, and drug candidate screening were conducted as exploratory analyses to generate biological and therapeutic hypotheses for future investigation. These exploratory analyses were not considered evidence of causal molecular interactions or therapeutic efficacy. Through this integrated framework, the study aimed to evaluate the prognostic significance of H19 and provide biological context for its potential role in GC progression.

## 2. Materials and Methods

This meta-analysis occurred in strict compliance with the Preferred Reporting Items for Systematic Reviews and Meta-Analyses (PRISMA) criteria [22]. The completed PRISMA checklist is shown in Supplementary Table S1. Our work has been registered in the International prospective register of systematic reviews (PROSPERO, CRD420251241547). The meta-analysis showed the associations of H19 expression with prognosis and clinicopathological characteristics in GC. UALCAN and GEO datasets were used for external validation of H19 expression and its clinical associations, while qRT-PCR assessed H19 expression in GC cell lines. Functional enrichment, regulatory network, and drug prediction analyses were conducted separately as exploratory analyses to generate hypotheses for future investigation.

### 2.1. Literature Sources and Search Strategy

A comprehensive systematic search was performed in the databases, including PubMed, Cochrane Library, Scopus, Ovid, Embase, EBSCO, and Web of Science. Studies examining the association between lncRNA H19 and its expression, clinicopathological features, or prognostic significance in GC were identified. The search covered all articles published from the establishment of each database through October 2025. The search strategy combined medical subject headings and entry terms. The following terms were used: (“Neoplasms, Stomach” OR “Gastric Neoplasms” OR “Gastric Neoplasm” OR “Stomach Neoplasm” OR “Gastric Cancer” OR “Stomach Cancer” OR “Gastric tumor” OR “Stomach tumor” OR “Cancer of the Stomach” OR “Cancer of Stomach” OR “Gastric Cancer, Familial Diffuse”) AND (“H19” OR “H19 RNA” OR “H19 long non-coding RNA, human” OR “H19, imprinted maternally expressed transcript (non-protein coding), human” OR “lncRNA H19” OR “H19 long non-coding RNA”).

### 2.2. Inclusion and Exclusion Criteria

Inclusion criteria: (1) All included studies were required to be accessible through English databases, with complete and original research data. (2) Participants were categorized into high and low H19 expression groups using clearly defined experimental methods. (3) The study showed the prognostic value or clinicopathological relevance of H19 in clinical GC patients. (4) Hazard ratio (HR) and 95% Confidence interval (CI) were either directly provided or could be extracted from Kaplan-Meier (KM) curves. (5) Patients have not received surgery, radiotherapy, chemotherapy, or anti-tumor drug treatment prior to the diagnosis of GC.

Exclusion criteria: (1) The research topic was irrelevant to lncRNA H19 and GC. (2) Duplicate articles. (3) Prognostic data obtained from the public TCGA datasets. (4) Article types such as reviews, case reports, meta-analyses, conference abstracts, letters, and retractions. (5) Non-human studies. (6) Clinical sample size fewer than 30 patients. (7) Studies lacking essential data.

### 2.3. Article Screening and Data Extraction

Pursuant to the predefined inclusion and exclusion criteria, relevant information from the final set of eligible studies was extracted. To ensure accuracy, two researchers independently screened the articles and performed data extraction. Disagreements were worked out by communication with a third researcher, who made the ultimate choice. The screening procedure commenced with an initial examination of titles and abstracts, succeeded by a comprehensive full-text evaluation of studies that appeared to fulfill the criteria. The extracted data encompassed author details, publication year, country, study duration, sample type, sample size, detection method, outcome, cut-off value, source of HR, and follow-up time. HR and 95%CI were obtained in two ways: (1) directly from the included studies that provided these values, (2) indirectly from studies that provided KM curves but not the values, which need to extract the data using Engauge Digitizer 11.1 software and key data were calculated in Excel 2021 [23].

### 2.4. Quality Assessment of Included Studies

Studies that satisfied the inclusion criteria were assessed with the Newcastle-Ottawa Scale (NOS) [24]. The NOS encompasses three core dimensions (selection, comparability, and exposure) that are split into eight sub-dimensions, with a maximum achievable score of 9 points. Research scoring 0–5 points were categorized as low quality, whereas those scoring 6–9 points were classified as high quality, and only high-quality studies were incorporated into the meta-analysis. To reduce subjective bias, two researchers conducted the quality evaluations separately; if there were any disagreements, they engaged in discussions or consulted a third researcher to reach a decisive decision.

### 2.5. External Validation of H19 Expression and Clinical Associations

To externally validate H19 expression and its clinical associations in GC samples, we utilized the University of Alabama at Birmingham Cancer Data Analysis Portal (UALCAN) (https://ualcan.path.uab.edu/ (accessed on 2 November 2025)) [25] database, and two RNA-seq datasets drawn from the Gene Expression Omnibus (GEO) (https://www.ncbi.nlm.nih.gov/geo/ (accessed on 2 November 2025)) [26] database (GSE208099 [27] and GSE6580 [28]). The UALCAN database was also employed to assess the prognostic value of H19 and its expression across different clinical stages of GC. Furthermore, the LinkedOmics database (https://www.linkedomics.org/login.php (accessed on 4 November 2025)) [29] was utilized to identify genes that exhibit positively and negatively correlated with H19, with the results visualized as heat maps.

### 2.6. Exploratory Functional and Regulatory Analyses of H19

To explore biological mechanisms potentially associated with H19, six analytical dimensions were included: Gene Ontology (GO) enrichment analysis, Kyoto Encyclopedia of Genes and Genomes (KEGG) pathway enrichment analysis, prediction of H19-targeted miRNAs and mRNAs, prediction of H19-related transcription factors (TFs), prediction of RNA-binding proteins (RBPs) interacting with H19, and prediction of drugs potentially targeting H19. The LinkedOmics database was used to identify genes positively and negatively correlated with H19 and to conduct GO and KEGG enrichment analyses. The ceRNA, TF, and RBP analyses explored potential post-transcriptional, transcriptional, and RNA-protein regulatory relationships, respectively, while drug preliminary prediction identified candidate compounds for further investigation. These analyses were exploratory and did not establish causal molecular interactions or therapeutic efficacy.

Prediction of H19 targeted miRNAs was performed using the ENCORI (https://rnasysu.com/encori/ (accessed on 11 November 2025)) [30], lnCAR (https://lncar.renlab.cn/#/home (accessed on 11 November 2025)) [31], LncACTdb3.0 (https://bio-bigdata.hrbmu.edu.cn/LncACTdb3.0/ (accessed on 11 November 2025)) [32] and RNAInter (http://www.rnainter.org/ (accessed on 11 November 2025)) [33], while miRNA targeted mRNAs were identified through LncACTdb3.0 and lnCAR. These results were integrated to construct the competing endogenous RNA (ceRNA) regulatory network involving H19, miRNAs, mRNAs. TFs related to H19 were predicted using the LncMAP (https://bio-bigdata.hrbmu.edu.cn/LncMAP/ (accessed on 12 November 2025)) [34] and RNAInter. Potential RBPs interacting with H19 were identified using RNAct (https://rnact.tartaglialab.com/ (accessed on 12 November 2025)) [35] and catRAPID (https://service.tartaglialab.com/page/catrapid_group (accessed on 12 November 2025)) [36]. Drug prediction analyses were conducted through RNAactDrug (http://bio-bigdata.hrbmu.edu.cn/RNAactDrug/ (accessed on 12 November 2025)) [37] and LncMAP to identify candidate therapeutic compounds associated with H19 regulation. All predicted interactions were ultimately visualized through Cytoscape software (version 3.9.1) [38] to generate an integrated interaction network.

### 2.7. Cell Culture and Quantitative Real-Time PCR (qRT-PCR) Analysis

Human gastric mucosal epithelial cell GES-1 and human GC cell lines (AGS and SGC7901) were obtained from the Chinese Academy of Sciences. GES-1 and SGC7901 cells were grown in RPMI1640 medium (Sangon Biotech, Shanghai, China), while AGS cells were maintained in F-12K medium (Boster, Wuhan, China). Each of these culture media was fortified with 10% fetal bovine serum and 1% penicillin/streptomycin. The cells were incubated at a constant temperature of 37 °C within a humidified incubator that contained 5% CO_2_.

Total RNA was obtained utilizing Trizol (Beyotime, Shanghai, China). cDNA was generated from total RNA through the BeyoRT™ II cDNA synthesis Kit (with gDNA Eraser) (Beyotime, Shanghai, China). qRT-PCR was executed employing the BeyoFast™ SYBR Green qPCR Mix (2×, High ROX) (Beyotime, Shanghai, China) on the ABI 7300 Real-Time PCR System (Thermo Fisher Scientific, Waltham, MA, USA). ACTB (Sangon Biotech, Shanghai, China) was used as the internal reference control to normalize H19 expression levels. Relative gene expression was determined via the 2^−ΔΔCt^ methodology. Primer sequences used for amplification are enumerated in Table 1.

### 2.8. Statistical Analysis

The meta-analysis was conducted using Stata 15 software. HR and 95% CI were pooled to evaluate the association between H19 expression and prognosis in GC patients. An HR greater than 1 with a 95% CI excluding 1 indicated that high H19 expression was associated with poorer prognosis. Given the anticipated clinical and methodological heterogeneity arising from differences in cut-off value, sample type, geographic settings, and methods of HR extraction, a DerSimonian-Laird random effects model was used as the primary analysis for OS. A fixed effect model was additionally applied as a sensitivity analysis to assess the robustness of the result to model choice. Heterogeneity was assessed using Cochran’s Q test and the I^2^ statistic, which were used to describe statistical heterogeneity rather than as the sole basis for model selection. Subgroup analyses were conducted according to sample size, follow-up duration, cut-off value, and NOS score. Leave one out sensitivity analysis was performed by sequentially excluding each study. Publication bias was assessed using Begg’s and Egger’s tests when at least ten studies were available, with *p* ≤ 0.05 indicating statistical significance. Regarding the qRT-PCR results, statistical analyses were performed using GraphPad Prism 9.0. Comparisons between two groups were analyzed using an unpaired *t*-test, whereas comparisons among multiple groups were conducted using one-way ANOVA. A *p* value below 0.05 was considered statistically significant. Statistical significance was indicated as follows: * *p* < 0.05; ** *p* < 0.01; *** *p* < 0.001; **** *p* < 0.0001.

## 3. Results

### 3.1. Article Retrieval Process

**Figure 1:**
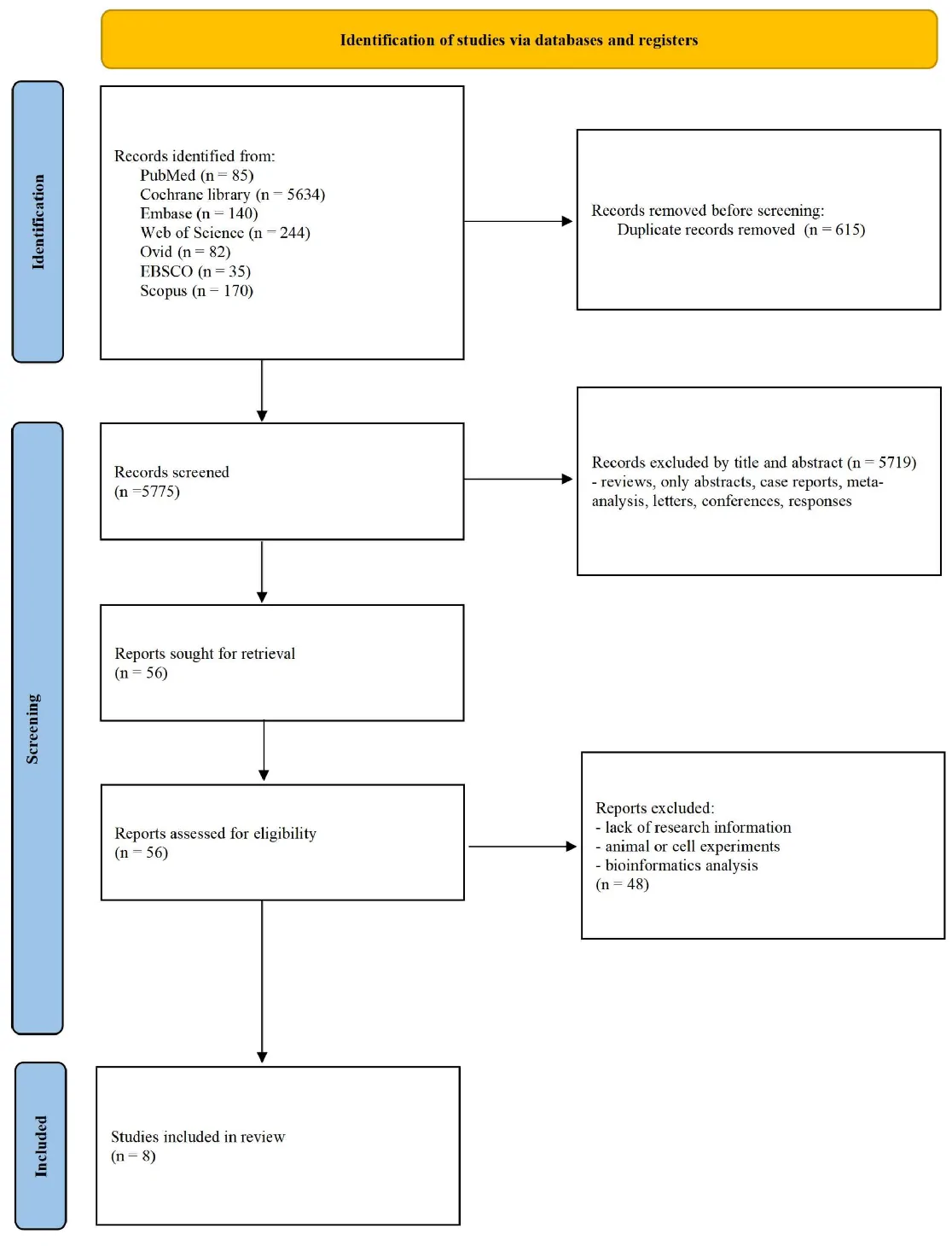
PRISMA flow chart of the article screening process.

Following the predetermined search strategy, a preliminary search of English online databases identified 6390 articles. Using EndNote X9 for initial screening combined with manual review, 601 duplicate articles were removed. Among the remaining 5789 articles, 5737 were subsequently excluded following an examination of their titles and abstracts, as they were categorized as reviews, abstracts, case reports, etc. The full texts of the 52 articles that remained were then subjected to a thorough evaluation, resulting in the exclusion of 44 articles due to insufficient or incomplete clinical prognostic data. In the end, 8 articles fulfilled the inclusion criteria and were incorporated into the meta-analysis, encompassing a total of 768 patients. The detailed screening strategy is shown in Figure 1.

### 3.2. Basic Information and Quality Evaluation (NOS Score) of the Included Studies

This meta-analysis encompassed 8 studies published from 2014 to 2025, with a combined participant pool of 768 individuals diagnosed with GC. Seven studies were conducted by Chinese researchers, and one study was published by a Turkish research team. All studies assessed H19 expression levels using qRT-PCR. Six of the eight studies reported specific cut-off values, including median, mean and fold-change thresholds, while two studies did not provide cut-off values. All included patients with GC had not received surgery, radiotherapy, chemotherapy, or any other treatments prior to diagnosis. Four studies divided patients into high- and low-H19 expression groups and reported detailed clinicopathological characteristics, whereas the remaining four studies did not provide such information. All eight included studies provided overall survival (OS) as the outcome, and one study also provided disease-free survival (DFS). The NOS was adopted to appraise the quality of the selected studies; with the resulting scores falling from 6 to 8, indicating that the quality was relatively high. The basic information and quality evaluations extracted from the eight articles are shown in Table 2.

### 3.3. Association Between H19 Expression Level and OS in GC Patients

**Figure 2:**
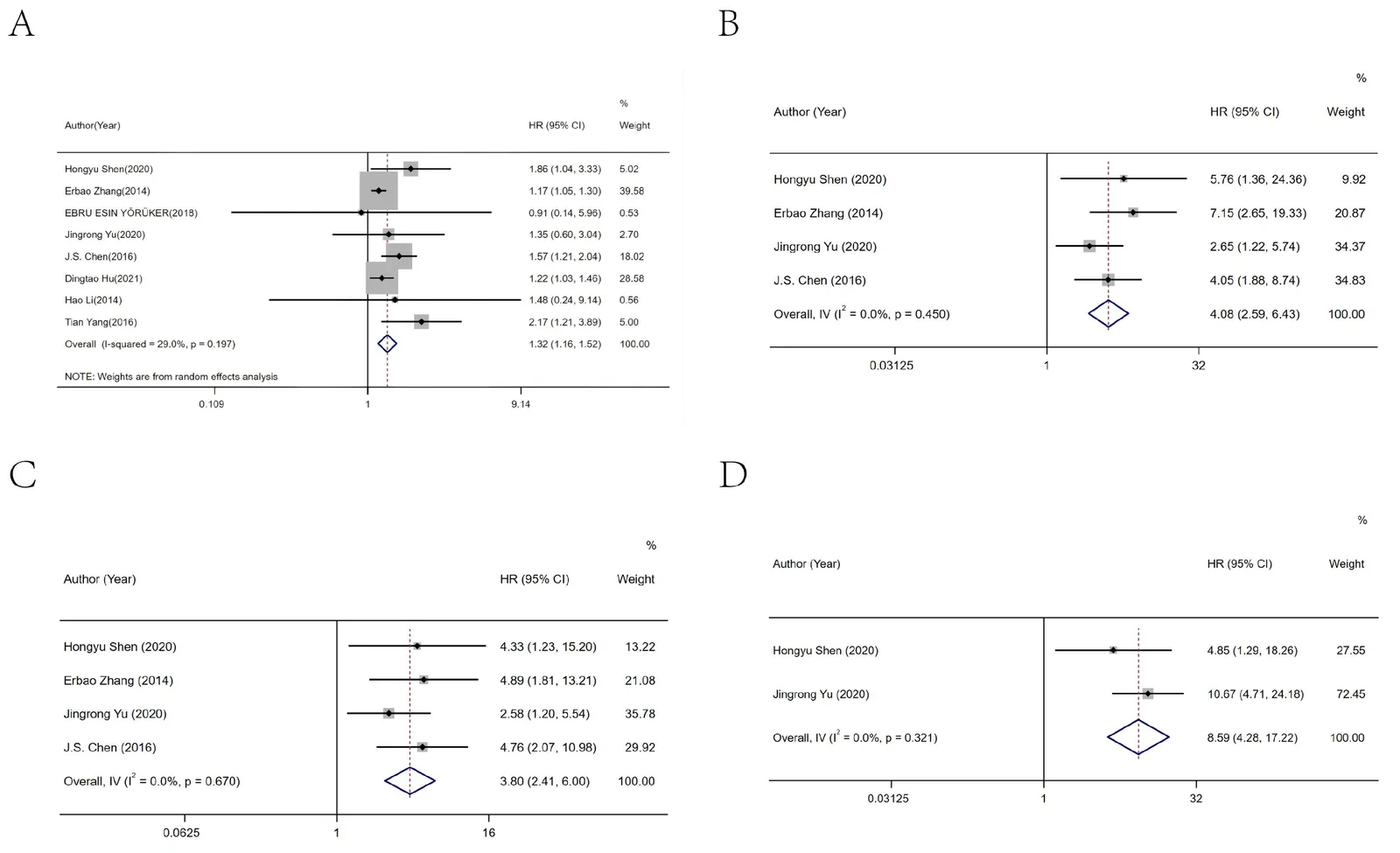
Forest plots of the correlation between H19 expression levels and GC patients. (**A**) OS [18,39,40,41,42,43,44,45]. (**B**) TNM stage in clinicopathological data [18,39,40,42]. (**C**) Lymph node metastasis in clinicopathological data [18,39,40,42]. (**D**) Tumor invasion in clinicopathological data [18,39].

All 8 included studies reported OS, and H19 expression levels were consistently higher in GC tissues compared to adjacent non-cancerous tissues. Using a random effects model, elevated H19 expression was significantly associated with poorer OS, with a combined HR of 1.32 (95% CI = 1.16–1.52; I^2^ = 29.0%, *p* = 0.197). Tissues with high H19-expressing gastric tumor was significantly linked to poorer OS (z = 4.02, *p* < 0.05), revealing that H19-high patients had a worse prognosis relative to those with H19-low (Figure 2A).

### 3.4. Relationship Between H19 Expression Levels and the Clinicopathological Characteristics of GC Patients

To explore the connection between H19 expression levels and clinicopathological characteristics in GC, we analyzed TNM (Tumor-Node-Metastasis) stage, lymph node metastasis, and tumor invasion based on data from the eight included studies. Among them, four studies reported TNM stage and lymph node metastasis, while only two studies provided information on tumor invasion. For TNM stage, pooled analysis of the four eligible studies showed a combined effect size of HR = 4.08, 95%CI = 2.59–6.43, and the heterogeneity test was I^2^ = 0.0%, *p* = 0.450 and a fixed-effect model was used. Higher H19 expression was substantially connected with more advanced TNM stage (z = 6.068, *p* < 0.05). The results are shown in Figure 2B. For lymph node metastasis, the gathered effect size was HR = 3.80, 95%CI = 2.41–6.00, and the heterogeneity test was I^2^ = 0.0%, *p* = 0.670, and a fixed-effect model was chosen. Higher H19 expression was notably correlated with lymph node metastasis (z = 5.732, *p* < 0.05), indicating that patients with elevated H19 expression levels were more likely to develop lymphatic spread. The results are shown in Figure 2C. For tumor invasion, only two studies provided relevant clinicopathological data. The findings proved the summed effect size was HR = 8.59, 95%CI = 4.28–17.22, and heterogeneity test was I^2^ = 0.0%, *p* = 0.321, and a fixed effect model was used. Elevated H19 expression was starkly correlated with tumor invasion (z = 6.053, *p* < 0.05), suggesting that high H19 expression levels may promote invasive tumor behavior. These results are shown in Figure 2D.

### 3.5. Subgroup Analysis

To further explore the prognostic value of H19 expression in patients with GC, random effects subgroup analyses were conducted according to sample size, follow-up time, cut-off value, and NOS score. For sample size, the combined HR was 1.19 (95%CI = 1.07–1.32) for studies with fewer than 100 participants and 1.44 (95%CI = 1.15–1.80) for studies with at least 100 participants. For follow-up time, the combined HR was 1.27 (95%CI = 0.98–1.63) for studies with follow-up of at least 40 months and 1.54 (95%CI = 1.20–1.97) for studies with shorter follow-up. The combined HR was 1.58 (95%CI = 1.26–1.98) for the median cut-off subgroup, whereas the combined HR was 1.17 (95%CI = 1.05–1.30) for the others cut-off value subgroup. For study quality, the combined HR was 1.45 (95% CI = 1.07–1.96) for studies with NOS scores of at least 7 and 1.34 (95% CI = 1.11–1.62) for studies with lower scores.

**Figure 3:**
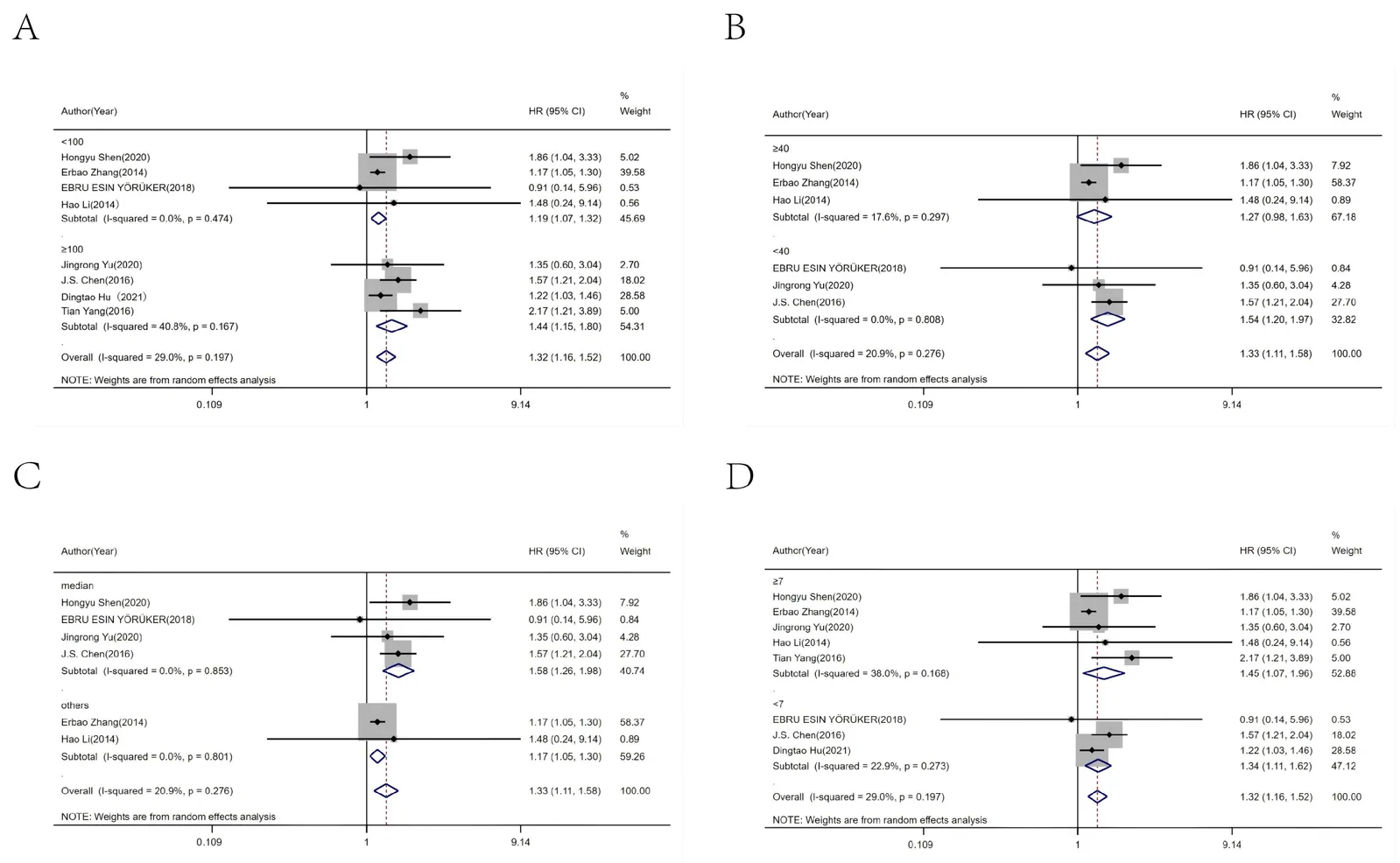
Subgroup analyses of the correlation between H19 expression levels and OS in GC patients. (**A**) Sample size [18,39,40,41,42,43,44,45]. (**B**) follow-up time [18,39,40,41,42,44]. (**C**) Cut-off value [18,39,40,41,42,44]. (**D**) NOS score [18,39,40,41,42,43,44,45].

Among the factors, the cut-off value was identified as a key methodological variable influencing the estimation of the prognostic effect, and therefore its subgroup analysis results warrant particular attention. The median cut-off group showed a combined effect size of HR = 1.58, 95%CI = 1.26–1.98, suggesting a strong association between high H19 expression and poor prognosis in GC patients. In comparison, the groups using other standard cut-off definitions showed an effect size of HR = 1.17, 95% CI = 1.05–1.30, indicating a relatively weaker effect than that observed in the median group. The heterogeneity of both groups remained within an acceptable range. Although the magnitude of the effect varied across the groups, the direction of the association remained entirely consistent, which effectively reduced the possibility of false positive conclusions caused by cutoff value selection bias. The association did not reach statistical significance in the subgroup with follow-up of at least 40 months. Moreover, subgroup analyses based on sample size, shorter follow-up time, and NOS score consistently demonstrated that elevated H19 expression remained stably associated with poor prognosis in GC. These findings further confirmed the robustness of the association between H19 expression and GC prognosis. The detailed results of each subgroup analysis are shown in Figure 3A–D.

### 3.6. Sensitivity Analysis and Publication Bias

**Figure 4:**
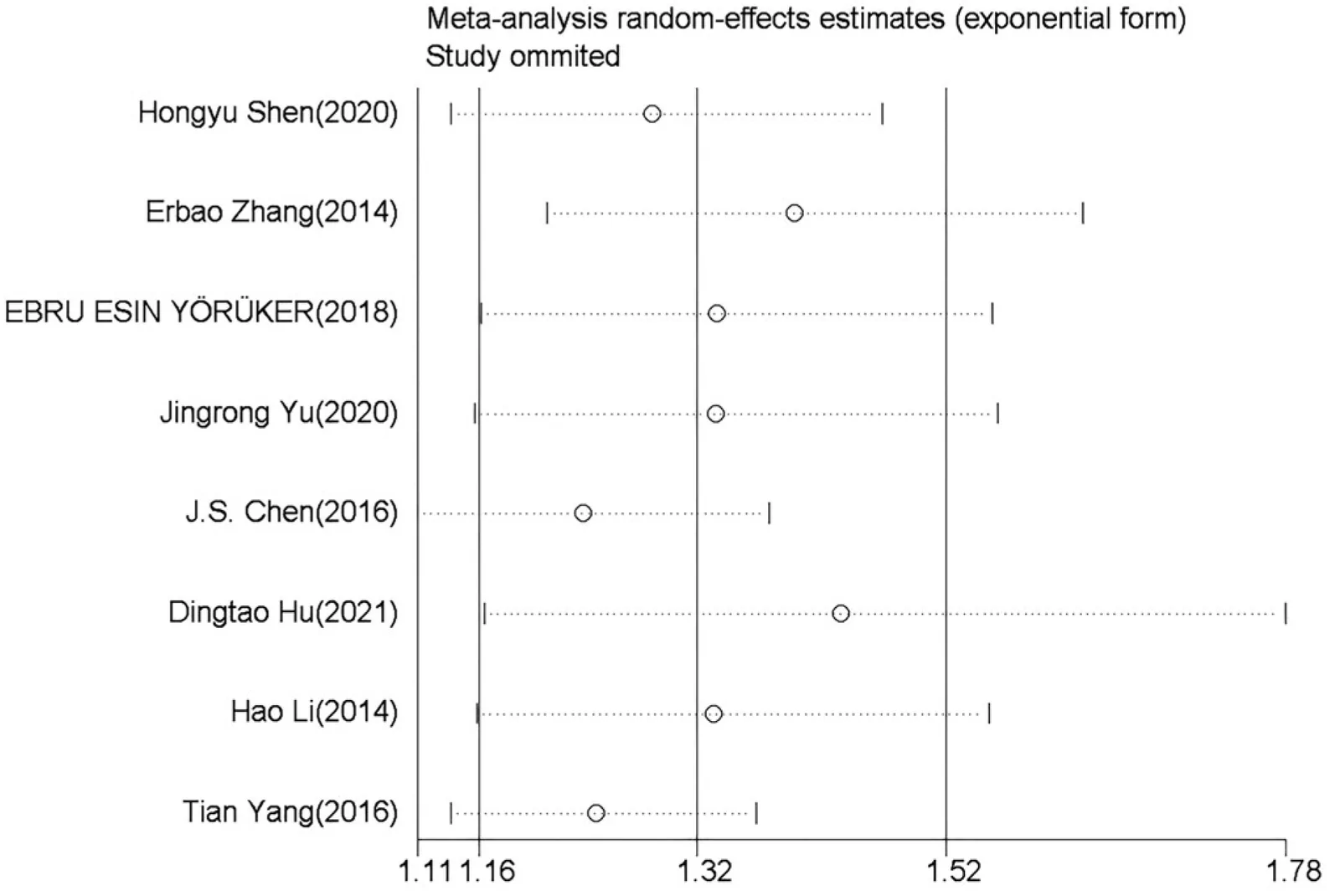
Sensitivity analysis [18,39,40,41,42,43,44,45].

To assess the robustness of the pooled OS result, sensitivity analysis was conducted by leaving one out and changing the model. The combined results remained statistically significant after exclusion of each individual study. This finding indicates that no single study materially altered the overall association (Figure 4). By changing the analysis model and using the fixed effect, the combined HR obtained was 1.25 (95% CI = 1.15–1.36). The combined results were consistent with the previous ones and were all statistically significant (z = 5.179, *p* < 0.05). The two sensitivity analysis methods show that the result is stable and reliable. The detailed data of the corresponding leave one out method and the result of the fixed effect model can be found in Supplementary Table S2 and Figure S1. Because fewer than ten studies were included, publication bias was not formally assessed.

### 3.7. External Validation of H19 Expression and Clinical Associations

**Figure 5:**
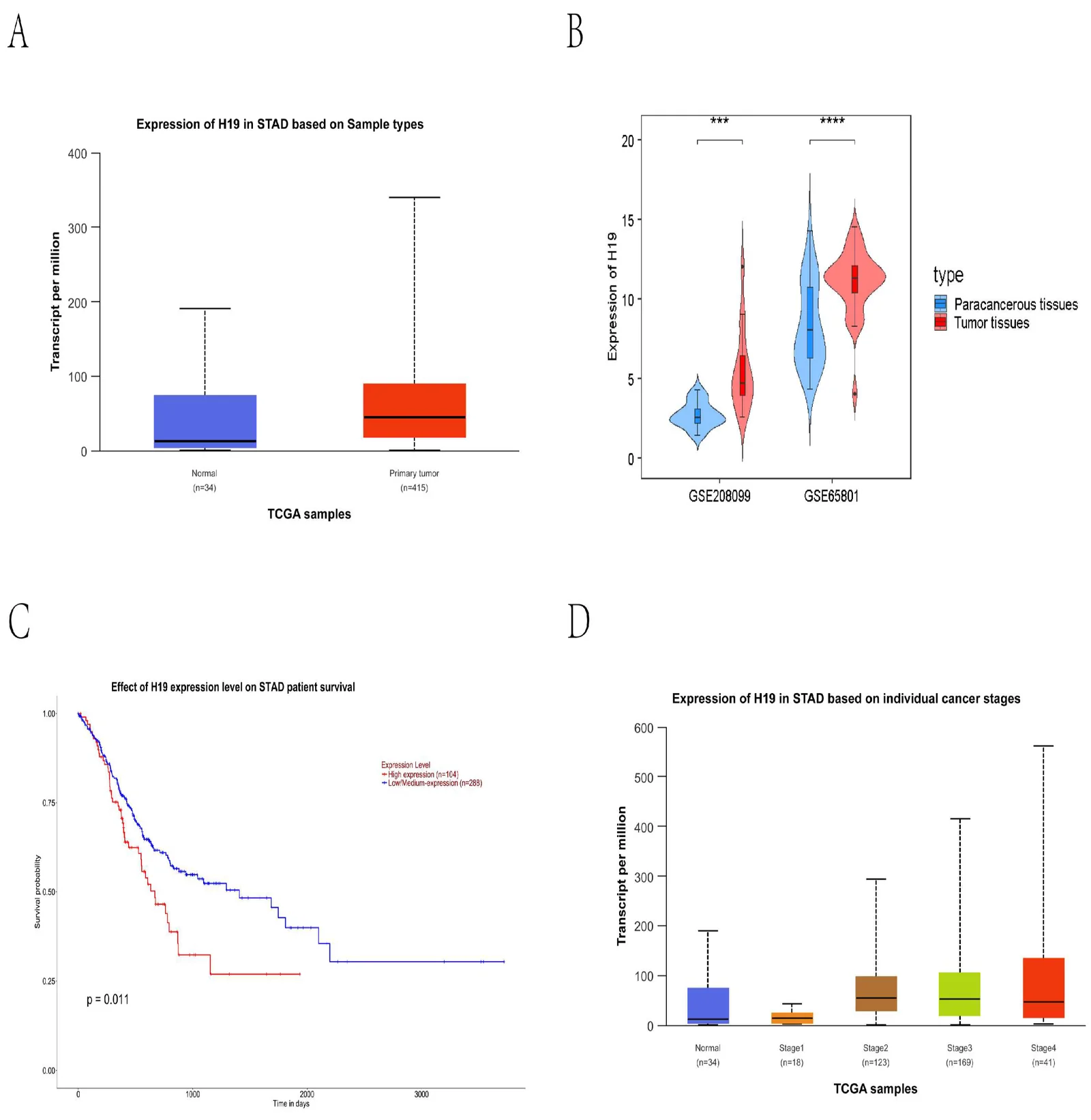
External Validation of H19 Expression and Clinical Associations. (**A**) Expression levels of H19 in normal controls and GC tissues using data from the UALCAN database. (**B**) H19 expression levels in adjacent normal tissues and cancer tissues in the GEO datasets GSE208099 and GSE65801. *** *p* < 0.001; **** *p* < 0.0001. (**C**) OS analysis of H19 in GC patients in the UALCAN database. (**D**) Expression levels of H19 in normal tissues versus GC patients’ tissues at different stages in the UALCAN database.

The UALCAN and GEO analyses were used to examine whether the expression and clinical associations of H19 identified in the meta-analysis could be reproduced in independent datasets. The UALCAN database and two GEO datasets, GSE208099 and GSE65801, were used to assess H19 expression levels. Raw data from the GEO datasets were acquired and analyzed for differential expression. H19 expression was significantly higher in GC tissues than in normal controls across UALCAN, GSE208099, and GSE65801 (*p* < 0.05; Figure 5A,B). UALCAN survival analysis showed that patients with high H19 expression had shorter OS (*p* < 0.05; Figure 5C), consistent with the meta-analytic finding for OS. H19 expression was also significantly elevated across all GC stages compared with normal controls, with higher levels in advanced stages than in early-stage disease (*p* < 0.05; Figure 5D). This pattern was consistent with the association between elevated H19 expression and advanced TNM stage observed in the meta-analysis.

### 3.8. Exploratory Functional, Regulatory, and Drug Prediction Analyses of H19

The analyses in this section were exploratory and were conducted to generate hypotheses regarding the biological functions, regulatory relationships, and potential therapeutic relevance of H19. They were not interpreted as confirmation of causal mechanisms, direct molecular interactions, or therapeutic efficacy.

**Figure 6:**
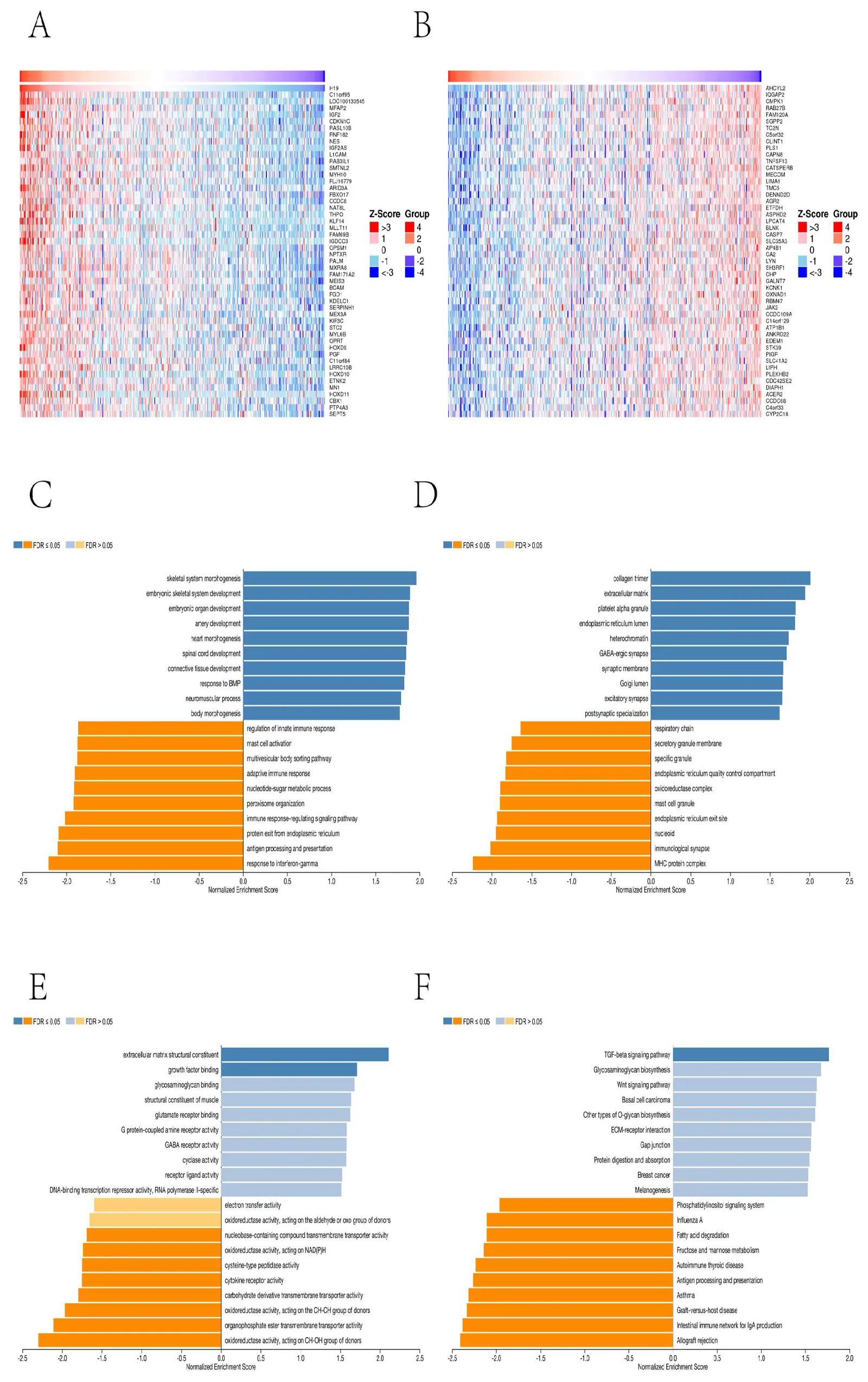
Exploratory GO and KEGG enrichment analyses of H19-related genes. (**A**) Heatmap of the top ten genes positively linked with H19. (**B**) Heatmap of the top ten genes negatively linked with H19. (**C**) BP category of GO enrichment analysis. (**D**) CC category of GO enrichment analysis. (**E**) MF category of GO enrichment analysis. (**F**) KEGG pathway enrichment analysis.

LinkedOmics identified genes correlated with H19 for subsequent exploratory analyses. The ten genes most positively correlated with H19 were C11orf95, LOC00133545, MFAP2, IGF2, CDKN1C, RASL10B, RNF182, NES, IGF2AS, and L1CAM, whereas the ten most negatively correlated genes were AHCYL2, IQGAP2, CMPK1, RAB27B, FAM120A, SGPP2, TC2N, C5orf32, CLINT1, and PLS1 (Figure 6A,B).

GO enrichment predictive analysis was conducted for H19 related genes from the three perspectives of BP, CC, and MF. In the BP category, H19 and its related genes were enriched in pathways involved in the skeletal system morphogenesis and showed significant negative correlations with the response to interferon-gamma (Figure 6C). In the CC category, these genes were predominately localized to the extracellular matrix and were involved in the formation of collagen trimer (Figure 6D). In the MF category, H19 associated proteins were enriched in extracellular matrix structural constituents and growth factor binding, consistent with their cellular localization. The negative regulation effect on oxidoreductase activity, acting on CH-OH group donors represented the most prominent enriched functions (Figure 6E). KEGG pathway enrichment predictive analysis revealed that H19 and its related genes may have a bearing on the emergence and development of GC mainly by regulating TGF-β signaling pathway. Among the predicted H19 interacting genes involved in this pathway, Inhibin Subunit Beta B (INHBB), Activin A Receptor Type 2B (ACVR2B) and Chordin (CHRD) showed the closest association. A literature search on Pubmed identified two GC-related studies on INHBB [46,47], whereas no research on ACVR2B or CHRD in GC has been reported to date, indicating potential novel targets for further investigations. Conversely, the most significantly negatively regulated predictive pathways associated with H19 and its related genes were allograft rejection and the intestinal immune network for lgA production, which aligns with the negative correlation observed for the interferon-gamma response in the BP analysis (Figure 6F). This can provide ideas for subsequent experimental research.

**Figure 7:**
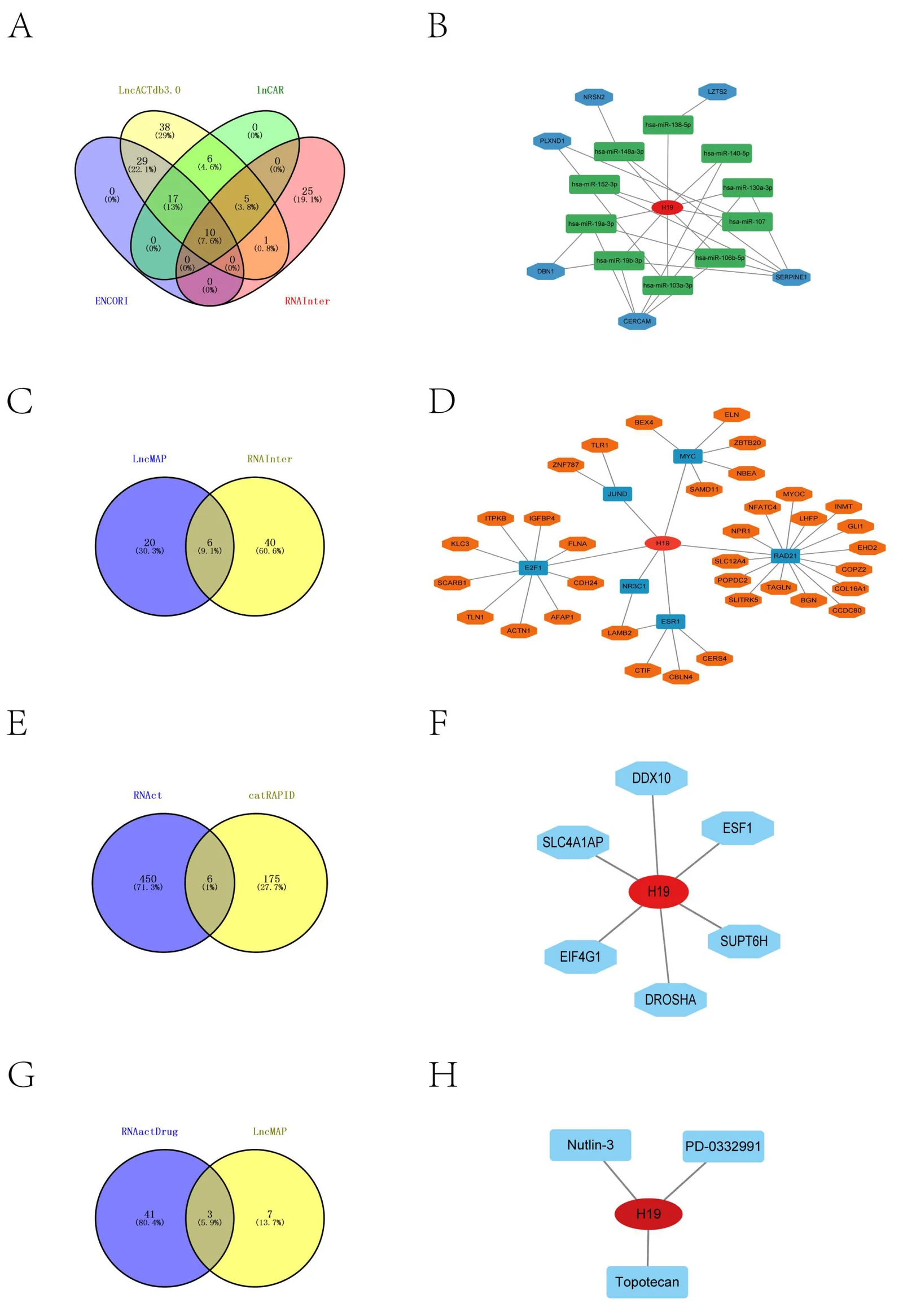
Exploratory prediction of H19 associated regulatory networks and candidate compounds. (**A**) Intersection diagram of H19-targeted miRNA prediction. (**B**) H19-miRNA-mRNA axis network diagram. (**C**) Intersection diagram of predicted TFs related to H19. (**D**) H19-TF-gene connection network diagram. (**E**) Intersection diagram of predicted RBPs interacting with H19. (**F**) H19-RBP interaction network diagram. (**G**) Intersection diagram of predicted drugs related to H19. (**H**) H19-drug interaction network diagram.

To further explore potential regulatory relationships involving H19 in GC, we predicted its related genes and constructed a network diagram to visualize the regulatory relationships within the gene axis. First, we identified miRNAs targeting H19 by intersecting results from multiple miRNA prediction databases, yielding 10 candidate miRNAs. Using the LncACTdb3.0 and lnCAR databases, we then predicted downstream mRNAs regulated through these miRNAs, resulting in six common target mRNAs: Drebrin 1 (DBN1), Serpin Family E Member 1 (SERPINE1), Plexin D1 (PLXND1), Cerebral Endothelial Cell Adhesion Molecule (CERCAM), Leucine Zipper Tumor Suppressor 2 (LZTS2), and Neurensin 2 (NRSN2). Among them, SERPINE1 and CERCAM showed the highest connectivity, each linked to five miRNAs, as shown in Figure 7A,B. Many lncRNAs exert regulatory effects on downstream mRNAs through transcriptional or post-transcriptional processes, thereby influencing the occurrence and development of diseases [48]. Dysregulation of TFs is known to affect processes such as breast cancer metastasis [49], chemotherapy resistance in colorectal cancer [50], and angiogenesis in clear cell renal cell carcinoma [51]. Therefore, we further predicted H19 associated TFs using the LncMAP and RNAInter databases and constructed the corresponding downstream regulatory network. A total of six TFs were identified, among which the RAD21 cohesin complex component (RAD21) exhibited the highest number of downstream target proteins (15 proteins). Additionally, Estrogen receptor 1 (ESR1) and Nuclear receptor subfamily 3 group C member 1 (NR3C1) shared a common downstream protein, laminin subunit beta 2 (LAMB2), as shown in Figure 7C,D. LncRNAs can serve as a molecular scaffolds, enabling RBPs in binding to mRNAs, thereby modulating mRNA stability and influencing disease progression. They can also regulate nuclear cytoplasmic localization and m6A modification, ultimately activating or inhibiting downstream proteins [52]. To investigate potential post-transcriptional regulators of H19, we predicted H19 associated RBPs and identified six RBPs potentially associated with H19, which were DEAD-Box helicase 10 (DDX10), ESF1 Nucleolar Pre-RRNA Processing Protein Homolog (ESF1), SPT6 homolog, histone chaperone and transcription elongation factor (SUPT6H), drosha ribonuclease III (DROSHA), eukaryotic translation initiation factor 4 gamma 1 (EIF4G1), solute carrier family 4 member 1 adaptor protein (SLC4A1AP). These predicted results are shown in Figure 7E,F. We further screened compounds potentially associated with H19 using the RNAactDrug and LncMAP databases. Nutlin-3, PD-0332991, and Topotecan were identified as candidate compounds for future experimental evaluation. These computational predictions do not demonstrate direct targeting of H19, inhibition of H19 related pathways, or therapeutic efficacy. The results are shown in Figure 7G,H.

### 3.9. Expression of H19 in GC Cells

**Figure 8:**
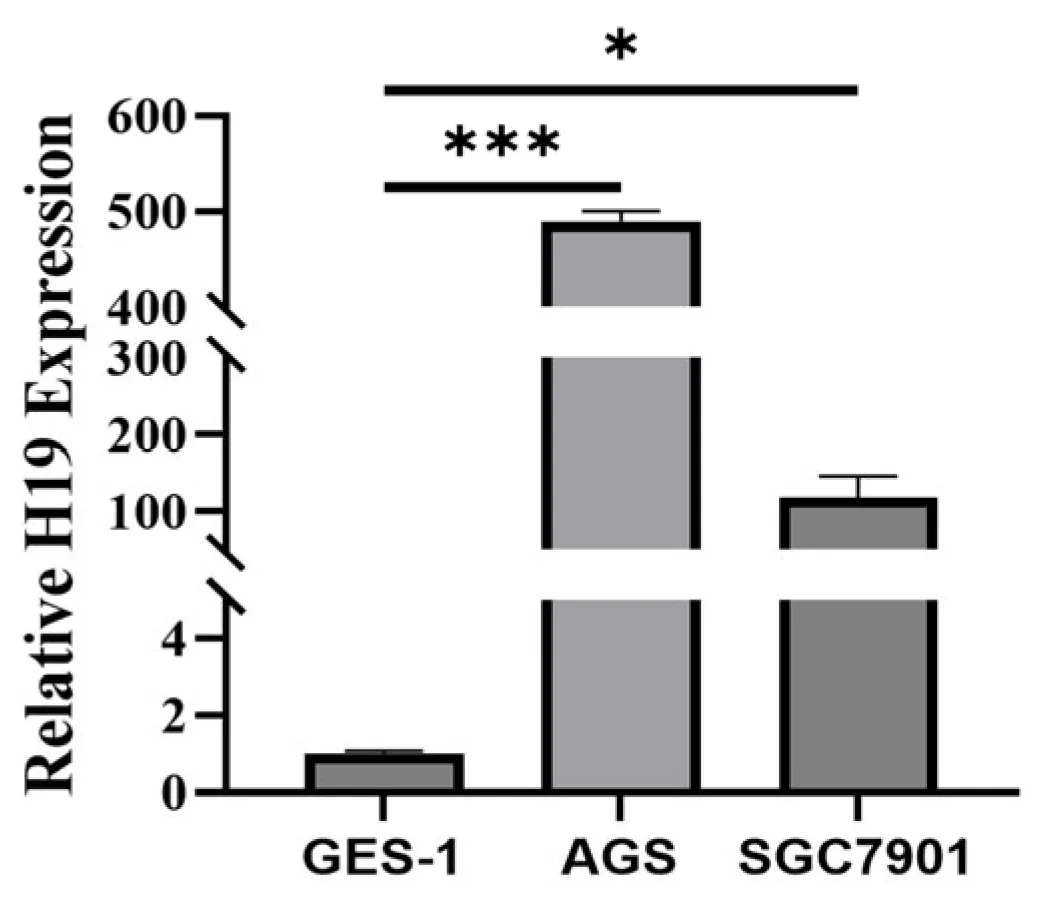
Expression levels of H19 in normal gastric mucosal epithelial cell GES-1 and GC cells. * *p* < 0.05; *** *p* < 0.001.

To ascertain the expression level of H19 in GC, we conducted qRT-PCR analysis on GC cancer cells. Compared to the normal cell (GES-1), the GC cells (AGS and SGC7901) showed extremely increased H19 expression (Figure 8). This result is consistent with the findings obtained from the differential expression analysis in the UALCAN and GEO database.

## 4. Discussion

Non-coding RNAs have emerged as a noteworthy biomarker in recent years, and the uncovering of lncRNAs has initiated a new chapter in the field of molecular and cellular biology. Due to their unique biological functions, lncRNA have become a major focus of current research. LncRNAs are endogenous transcripts lacking a functional open reading frame and were previously misunderstood as “transcriptional noise”. However, accumulating evidence shows that lncRNAs function as multi-level regulators, participating in gene activation and silencing, alternative mRNA splicing, protein translation and modification, intracellular transport, maintenance of mRNA stability, and chromatin remodeling [53,54]. Early studies on lncRNAs reveal that they can regulate tumor proliferation, migration and invasion by acting as ceRNAs, sponging miRNAs to regulate downstream mRNA expression. In addition, lncRNAs influence tumor occurrence and progression through methylation-mediated regulatory mechanisms. Many lncRNAs are upregulated in cancers, suggesting roles in chemotherapy resistance and inhibition of ferroptosis. Notably, lncRNA nomenclature often reflects disease association, cellular localization, or the position of surrounding genes, underscoring their high tissue specificity and relative stability. These characteristics make lncRNAs promising biomarker candidates for cancer diagnosis and prognosis.

In this meta-analysis, eight studies were included to evaluate the prognostic value of H19 expression in patients with GC. The primary random effects analysis showed that elevated H19 expression was significantly associated with shorter OS, with a combined effect size of HR = 1.32 (95% CI = 1.16–1.52). The UALCAN survival analysis provided external validation of the association between elevated H19 expression and shorter OS. Only one study provided DFS; thus, a meta-analysis of DFS could not be conducted, and the association between H19 expression and DFS could not be reliably interpreted. Given the available clinicopathological data, we further analyzed TNM stage, lymph node metastasis, and tumor invasion. These associations suggest that elevated H19 expression is linked to more aggressive clinicopathological features. However, the available data cannot determine its effects on treatment response. A random effects model was selected as the primary approach for OS because differences in cut-off value, sample types, geographic settings, and methods of HR derivation suggested that the included studies might estimate related but nonidentical effects. The low observed I^2^ was not considered sufficient evidence for assuming a single common effect, particularly given the small number of studies. Subgroup analysis by sample size, cut-off value, shorter follow-up time and NOS score did not affect the significant association between evaluated H19 expression and poor OS. However, in the subgroup with a follow-up time of at least 40 months, this association did not reach the statistical significance level. Notably, the cut-off value, as a bridge linking molecular expression levels with clinical outcomes, represents an important methodological variable that influences both the reproducibility and clinical applicability of molecular biomarkers. Regarding study reproducibility, the median cut-off value is based on the relative ranking of expression levels within the sample and is therefore less affected by extreme values, qRT-PCR detection systems, or baseline sample composition. As a result, it demonstrates greater stability and replicability across different laboratories and study cohorts. In contrast, the mean cut-off value is highly sensitive to extreme values, while the fold change cut-off value depends on the selection criteria of the control group. Consequently, both mean and fold change are more susceptible to variations in the study design and experimental conditions, which may lead to considerable variations in effect sizes for the same biomarker across studies. Nevertheless, all cut-off subgroups reached consistent conclusions, further supporting the robustness of the association between high H19 expression and the poor prognosis in GC. From the perspective of clinical applicability, establishing a uniform and clearly defined cut-off value selection is essential for translating molecular markers from basic research into routine clinical practice. Our findings suggest that the median cut-off value is easy to implement and provides stable results, making it potentially more suitable as a standardized criterion for the initial clinical application of H19. In contrast, other cut-off methods currently lack a unified standard, which may limit the comparability and recognition of detection results across different hospitals. This seriously hinders the clinical transformation of H19 as a prognostic marker. The sensitivity analysis conducted by leaving one out showed that after excluding each individual study, the magnitude of the aggregated data was slightly different, but no single study significantly changed the overall association, and it still had statistical significance. Using fixed effects model analysis to get the combined HR = 1.25 (95% CI = 1.15–1.36) showed that the correlation direction and statistical significance were consistent in each model; two methods of sensitivity analysis demonstrated the result is stable and reliable. Overall, high expression of H19 appears to be a potential risk component for poor survival in GC patients and might act as an advantageous prognostic biomarker. As nearly as we know, this is the first meta-analysis that systematically evaluates the prognostic significance of H19 expression in GC.

Previous experimental studies have reported that H19 may promote the development and progression of GC through several molecular mechanisms. Liu et al. [55] found that H19 enhances cell proliferation and invasion capabilities by interacting with miR-675 and identified RUNX1 as a downstream target. In this study, H19 expression was positively correlated with miR-675, while RUNX1, typically considered a tumor suppressor, was downregulated, ultimately activating the AKT/mTOR pathway to promote proliferation and invasion in AGS cells. Building on this, Yang et al. [56] further demonstrated that ectopic expression of H19 in AGS cells stimulates proliferation, whereas H19 knockdown induces apoptosis. Their immunoprecipitation experiments confirmed a interaction between H19 and p53, indicating that H19 silencing leads to partial activation of p53. Liu et al. [57] research revealed that curcumin inhibits the proliferation of SGC-7901 cells in a concentration-dependent manner, accompanied by upregulation of p53 and downregulation of H19. Overexpression of H19 reversed these effects, and the authors identified c-myc as a mediator linking curcumin to H19 regulation. These findings suggest that curcumin suppresses the proliferation of SGC7901 and induces apoptosis through the c-myc/H19 axis. Zhang et al. [58] noticed that H19 upregulation in GC patients with Helicobacter pylori infection. Evaluated H19 expression increased the expression levels of inflammatory factors (TNF-α, IL-1β, and IL-8), and promoted GC cell proliferation, migration and invasion via the activation of NF-κB pathway. Gan et al. [59] established that high expression of H19 is tightly related to the TNM stage and lymph node metastasis. Mechanistically, H19 regulates the EMT and stimulates GC metastasis by targeting miR-22-3p to upregulate Snail1. These mechanistic observations derive from previous experimental studies and were not directly evaluated in the present meta-analysis. Most published studies have focused on individual molecular pathways and have not systematically linked H19 ex-pression with clinical outcomes. The present meta-analysis instead pooled clinical evidence for the statistical associations of H19 expression with OS, TNM stage, lymph node metastasis, and tumor invasion. The exploratory GO and KEGG, ceRNA, TF, and RBP analyses further identified candidate pathways and regulatory relationships for future investigation. However, these predictions do not demonstrate synergistic effects across pathways or establish causal links between the proposed mechanisms and prognosis. Accordingly, the present findings support further investigation of the relationships among H19 biology, tumor phenotype, and clinical outcomes rather than establishing a validated mechanistic framework.

Although this study has several strengths, including a detailed search process and rigorous methodology, it also has some limitations. (1) The majority of the included studies were conducted in China, with only one study from Turkey, while data from other ethnic groups and regions were lacking. Differences in genetic background, GC characteristics, and treatment strategies across populations may affect the applicability of the findings. Therefore, the limited ethnic and regional diversity of the included studies may restrict the generalizability of our conclusions to other populations worldwide. (2) Reported cut-off values varied notably among studies (mean, median, fold change), resulting in a lack of standardization and potential inconsistencies in the results. (3) Some of the included studies did not directly report HR and 95%CI, but instead provided KM survival curves. In these cases, HR and 95%CI were indirectly estimated using widely recognized methods. Although this method is considered the standard method for handling missing prognostic data in meta-analyses, indirectly extracted data inevitably introduce potential bias compared with directly reported results from the original studies. For example, technical limitations during the data extraction process, such as image resolution and overlap of KM curves, may affect extraction accuracy. In addition, subjective differences among operators may introduce random errors. Moreover, KM curves only provide cumulative survival rates at different time points and cannot fully reflect details such as the exact time and distribution of lost cases in the study, the characteristics of deleted data, or potential multivariate adjustment information from the original studies. Although we strictly adopted independent data extraction to minimize human error and further evaluated the robustness of the combined results through sensitivity analysis, the potential bias caused by these methodological limitations could not be completely eliminated. Therefore, we hope that future studies will prioritize the direct reporting of prognostic data to improve reliability and accuracy of subsequent meta-analyses. (4) The limited sample size of the included studies may increase random errors among studies, contribute to potential statistical heterogeneity, reduce the statistical validity of the analysis, and potentially limit the ability to detect weak associations. (5) The present analysis only focused on OS, thereby limiting the prognostic evaluation to a single clinical outcome. The prognostic value of H19 in other important aspects of GC, such as recurrence and disease progression, could not be comprehensively assessed. (6) While all studies used qRT-PCR to detect H19 expression, differences in primer sequences could contribute to heterogeneity. (7) This meta-analysis, by integrating data from multiple studies, demonstrated an association between H19 expression levels and poor prognosis in GC. However, we acknowledge several limitations regarding the causal inference capability of this study. Specifically, a mendelian randomization based causal inference framework, including genome-wide association studies (GWAS), expression quantitative trait locus (eQTL), and instrumental variable approaches, was not applied. As demonstrated by Zhang et al. [60], integrating eQTL data with GWAS prognostic data and utilizing genetic variants as instrumental variables can effectively reduce common confounding biases in traditional epidemiological or transcriptomic association studies, such as tumor microenvironment heterogeneity and differences in underlying disease status. In addition, this approach can minimize the effects of horizontal pleiotropy and reverse causal association. (8) The GO and KEGG enrichment, ceRNA, TF, RBP, and drug prediction analyses were computational and exploratory. The predicted pathways, regulatory relationships, and candidate compounds require experimental and clinical validation and should not be interpreted as established mechanisms or therapeutic options. These limitations should be considered when interpreting the magnitude, precision, generalizability, and clinical implications of the present findings.

Future research should include larger, multiethnic cohorts from different countries and consider multiple prognostic endpoints, such as DFS and progression-free survival (PFS), rather than relying solely on OS. Although OS is considered the gold standard for prognostic assessment, it can be significantly influenced by subsequent treatments and non-tumor-related deaths, thereby limiting its ability to accurately reflect the impact of H19 on the biological behavior of tumors. DFS is particularly applicable to early-stage GC patients who have undergone radical resection and may directly assess the predictive value of H19 expression for postoperative tumor recurrence. PFS is more suitable for patients with advanced, unresectable, or metastatic GC, as it can better reflect the effect of H19 on tumor growth, invasion, and chemotherapy resistance. Incorporating these endpoints would enable a more comprehensive evaluation of the prognostic value of H19 across different clinical settings. For genetic causal validation, GWAS data for GC and eQTL data from corresponding tissues were integrated to perform MR analyses based on summary data, aiming to elucidate the causal relationship between H19 expression and GC risk. At the single-cell level, approaches such as the “Scissor” algorithm described by Zhang et al. [61] could be used to identify specific cell populations associated with H19 ex-pression and generate more precise hypotheses regarding its role in the tumor micro-environment.

## 5. Conclusions

The primary random effects analysis showed that higher H19 expression in GC patients is significantly connected with poorer OS. Clinicopathological analysis revealed that H19 upregulation was associated with advanced TNM stage, lymph node metastasis, and tumor invasion, suggesting its potential prognostic value in GC. The exploratory bioinformatics analyses generated hypotheses regarding potential regulatory mechanisms and candidate compounds, but these findings require experimental and clinical validation. Further well-designed, large-scale, multinational studies incorporating multiple survival endpoints are needed to confirm the prognostic value and clinical applicability of H19.

## Figures and Tables

**Table 1:** Forward and reverse primer sequences.

Gene	Forward Primer Sequences	Reverse Primer Sequences
ACTB	CCTGGCACCCAGCACAAT	GGGCCGGACTCGTCATAC
H19	CAGGAGTGATGACGGGTGGAG	CTTCTTTCCAGCCCTAGCTCA

**Table 2:** Basic information of all eligible studies included in this meta-analysis.

Author	Year	Country	Study Duration	Sample Size (*n*)	Sample Type	Detection Method	Cut Off Value	Outcome	Source of HR	Follow-Up Months	NOS Scores
Hongyu Shen [39]	2020	China	NR	50	Tissue	qRT-PCR	median ratio	OS	②	63	8
ErBao Zhang [40]	2014	China	2006–2007	80	Tissue	qRT-PCR	mean	OS	①	60	7
EBRU ESIN YÖRÜKER [41]	2018	Turkey	2014–2016	41	plasma	qRT-PCR	median ratio	OS	②	44	6
Jingrong Yu [18]	2020	China	2015–2016	112	Tissue	qRT-PCR	median ratio	OS	②	36	7
J. S. CHEN [42]	2016	China	2013–2014	128	Tissue	qRT-PCR	median ratio	OS/DFS	①	36	6
Dingtao Hu [43]	2021	China	NR	150	blood	qRT-PCR	NR	OS	①	NR	6
Hao Li [44]	2014	China	2009–2011	74	Tissue	qRT-PCR	fold change	OS	②	53	8
Tian Yang [45]	2016	China	2014–2015	133	serum	qRT-PCR	NR	OS	①	NR	8

HR (hazard ratio); NR (not reported); OS (overall survival); DFS (disease-free survival); ① (directly provided); ② (survival curve).

## Data Availability

The original data provided in this study can be obtained from the corresponding author.

## Supplementary Materials

The following supporting information can be downloaded at: https://www.mdpi.com/article/10.3390/ijms27209276/s1.

## Abbreviations

The following abbreviations are used in this manuscript:

GC	Gastric cancer
cricRNA	circular RNA
lncRNA	Long noncoding RNA
miRNA	MicroRNA
H19	LncRNA H19
EMT	Epithelial–mesenchymal Transition
PRISMA	Preferred Reporting Items for Systematic Reviews and Meta-Analyses
PROSPERO	International prospective register of systematic reviews
HR	Hazard ratios
CI	Confidence intervals
KM	Kaplan-Meier
NOS	Newcastle-Ottawa Scale
UALCAN	University of ALabama at Birmingham Cancer Data Analysis Portal
GEO	Gene Expression Omnibus
GO	Gene Ontology
KEGG	Kyoto Encyclopedia of Genes and Genomes
TFs	Transcription factors
RBPs	RNA-binding proteins
CC	Cellular component
MF	Molecular function
BP	Biological process
ceRNA	Competing endogenous RNA
OS	Overall survival
DFS	Disease-free survival
TNM	Tumor-Node-Metastasis
DBN1	Drebrin 1
SERPINE1	Serpin Family E Member 1
CERCAM	Cerebral Endothelial Cell Adhesion Molecule
NRSN2	Neurensin 2
LZTS2	Leucine Zipper Tumor Suppressor 2
PLXND1	Plexin D1
RAD21	RAD21 cohesin complex component
NR3C1	Nuclear receptor subfamily 3 group C member 1
ESR1	Estrogen receptor 1
LAMB2	Laminin subunit beta 2
DDX10	DEAD-Box helicase 10
ESF1	ESF1 Nucleolar Pre-RRNA Processing Protein Homolog
SUPT6H	SPT6 homolog, histone chaperone and transcription elongation factor
DROSHA	Drosha ribonuclease III
EIF4G1	Eukaryotic translation initiation factor 4 gamma 1
SLC4A1AP	Solute carrier family 4 member 1 adaptor protein
GWAS	Genome-wide association studies
eQTL	expression quantitative trait locus
