# Supplementary material for: Prognostic Value of lncRNA H19 in Gastric Cancer: A Systematic Review and Meta-Analysis
Source: Int J Mol Sci. 2026 Oct 19;27(20):9276. doi: 10.3390/ijms27209276 (PMC13705260; doi:10.3390/ijms27209276)
Supplement: Supplementary file 1 [file ijms-27-09276-s001.zip › Supplementary Materials/Supplementary Figure S1.pdf]

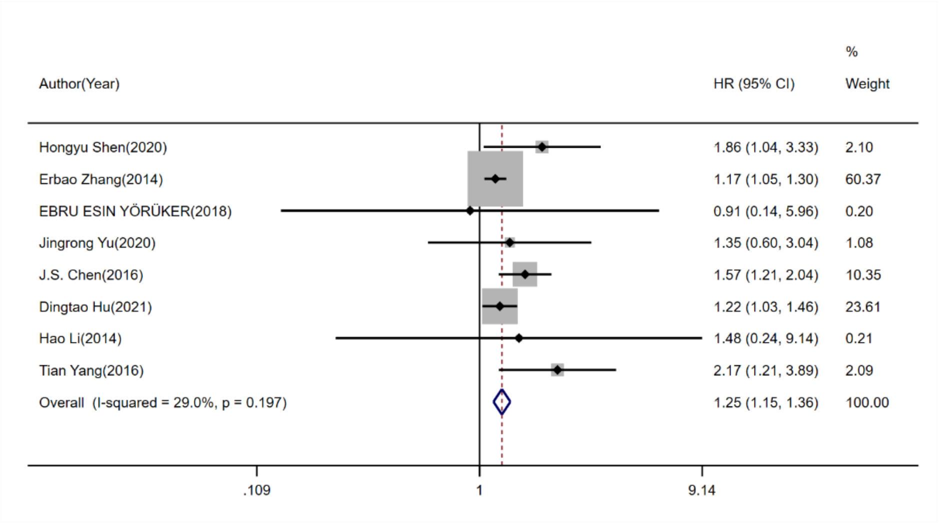

Supplementary Figure S1.Forest plot of fixed effect mode.
