# Supplementary material for: Prognostic Value of lncRNA H19 in Gastric Cancer: A Systematic Review and Meta-Analysis
Source: Int J Mol Sci. 2026 Oct 19;27(20):9276. doi: 10.3390/ijms27209276 (PMC13705260; doi:10.3390/ijms27209276)
Supplement: Supplementary file 1 [file ijms-27-09276-s001.zip › Supplementary Materials/Supplementary Table S2.pdf]

Supplementary Table S2. Detailed data of sensitivity analysis for leaving one out.

Analysis	omitted study	No. of studies	HR (95% CI)	I^2 (%)	*p* value
Leave one out sensitivity analysis	Hongyu Shen (2020) omitted	7	1.29 (1.13–1.47)	25.1	0.237
	Erbao Zhang (2014) omitted	7	1.40 (1.21–1.63)	5.2	0.387
	EBRU ESIN YÖRÜKER (2018) omitted	7	1.34 (1.16–1.56)	38.5	0.135
	Jingrong Yu (2020) omitted	7	1.34(1.15–1.56)	38.9	0.132
	J.S. Chen (2016) omitted	7	1.24 (1.11–1.38)	8.4	0.364
	Dingtao Hu (2021) omitted	7	1.44 (1.16–1.78)	38.7	0.134
	Hao Li (2014) omitted	7	1.34 (1.15–1.55)	39.0	0.132
	Tian Yang (2016) omitted	7	1.24 (1.13–1.37)	5.3	0.387

Note: HR, hazard ratio; CI, confidence interval.
